# Head-orienting behaviors during simultaneous speech detection and localization

**DOI:** 10.3389/fpsyg.2024.1425972

**Published:** 2024-09-17

**Authors:** Angkana Lertpoompunya, Erol J. Ozmeral, Nathan C. Higgins, David A. Eddins

**Affiliations:** ^1^Department of Communication Sciences and Disorders, University of South Florida, Tampa, FL, United States; ^2^Department of Communication Sciences and Disorders, Faculty of Medicine, Ramathibodi Hospital, Mahidol University, Bangkok, Thailand; ^3^Department of Communication Sciences and Disorders, University of Central Florida, Orlando, FL, United States

**Keywords:** head movements, visual cues, spatial cues, masking, speech detection, dual-task

## Abstract

Head movement plays a vital role in auditory processing by contributing to spatial awareness and the ability to identify and locate sound sources. Here we investigate head-orienting behaviors using a dual-task experimental paradigm to measure: (a) localization of a speech source; and (b) detection of meaningful speech (numbers), within a complex acoustic background. Ten younger adults with normal hearing and 20 older adults with mild-to-severe sensorineural hearing loss were evaluated in the free field on two head-movement conditions: (1) head fixed to the front and (2) head moving to a source location; and two context conditions: (1) with audio only or (2) with audio plus visual cues. Head-tracking analyses quantified the target location relative to head location, as well as the peak velocity during head movements. Evaluation of head-orienting behaviors revealed that both groups tended to undershoot the auditory target for targets beyond 60° in azimuth. Listeners with hearing loss had higher head-turn errors than the normal-hearing listeners, even when a visual location cue was provided. Digit detection accuracy was better for the normal-hearing than hearing-loss groups, with a main effect of signal-to-noise ratio (SNR). When performing the dual-task paradigm in the most difficult listening environments, participants consistently demonstrated a wait-and-listen head-movement strategy, characterized by a short pause during which they maintained their head orientation and gathered information before orienting to the target location.

## 1 Introduction

In natural communication settings, listeners adjust to sound sources and their locations by orienting their head and eyes either to optimize signal reception or to provide socially accepted non-verbal cues (Higgins et al., 2023; Valzolgher, 2024). However, investigations of speech perception and localization in the free field are typically designed to control extraneous variables, like head movements, an approach that often sacrifices real-world validity. When assessing audition in the sound field, for example, head movement is typically minimized (e.g., via instruction or restraints) to reduce influences of changes to binaural cues, like interaural time differences and interaural level differences (Blauert, 1969; for review see Risoud et al., 2018). Consequently, much of our understanding of spatial hearing and speech-in-noise listening may miss the true effects seen when listeners are free to move their head and eyes to improve spatial awareness and speech understanding in complex acoustic environments.

In the earliest studies on the relationship between head movement and sound localization, Wallach (1940) designed a series of experiments that sought to isolate the proprioceptive effects of head movement from vestibular and visual feedback by simulating head movement with a swivel-chair and revolving screen. Subsequent experiments that assessed performance differences during head fixed vs. head free conditions, in tasks such as speech reception or sound-source localization showed a benefit of head free conditions (Kock, 1950; Thurlow and Runge, 1967; Perrett and Noble, 1997; Wightman and Kistler, 1999; Brimijoin et al., 2012; McAnally and Martin, 2014; Grange and Culling, 2016a,b). Thurlow and Runge (1967) for example, reported significantly lower error for locations in the horizontal plane when the head was free to move compared to a fixed-head condition, likely due to a decrease in front-back confusions. Perrett and Noble (1997) further showed that even small head movements can significantly improve localization accuracy. Their study revealed that listeners could use the information from head movements as small as 15 degrees to disambiguate front-back confusions. Wightman and Kistler (1999) also demonstrated that head movements provide a significant advantage in localizing sound sources in elevation. They found that listeners could accurately judge the elevation of a sound source when allowed to move their heads, but performed poorly when their heads were fixed. These are just a few of the studies that highlight the importance of head movements in sound source localization, and their role in providing a more accurate and robust perception of the auditory environment.

In addition to localization improvements with head movements, speech intelligibility can be improved during speech perception tasks that involve spatially separated target and masker streams. By orienting the head slightly away from the speech source, for example, listeners create a “head-orientation benefit” (Kock, 1950; Grange and Culling, 2016a). Grange and Culling (2016a) approximated this benefit to be about 8-dB when orientation was at an intermediate position between the target and masker locations. Head-orientation benefit is also demonstrated by the work of Brimijoin et al. (2012), who found that listeners asymmetric hearing loss naturally orient their heads to optimize the signal-to-noise ratio, thereby enhancing speech intelligibility. Moreover, the study by McAnally and Martin (2014) provides evidence that this behavior is not just a passive response, but an active strategy employed by listeners to improve their auditory perception.

The implications of head movements for listeners with hearing loss are significant and multifaceted. Speech intelligibility and localization accuracy are generally poorer for individuals with hearing loss compared to normal-hearing listeners, though studies often constrain head movements and perhaps overestimate real-world functional deficits (Noble et al., 1994; Lorenzi et al., 1999a,b; Kidd et al., 2015; Brimijoin and Akeroyd, 2016; Van den Bogaert et al., 2006). In studies that have attempted more ecological validity, those with hearing loss have been shown to have delayed head movement responses during sound localization tasks, and their head movement trajectories are significantly more complex when orienting to a target location (Brimijoin et al., 2010). Listeners with asymmetrical hearing loss also tend to turn their heads to maximize target speech level in their better ear, which does indeed result in increased speech intelligibility (Brimijoin et al., 2012; Gessa et al., 2022). This suggests that the disparity of auditory cues at the two ears can modulate the benefit of head movements. These representative studies (see Higgins et al., 2023 for further review) provide strong support for the importance of head movement to provide a better-ear and binaural unmasking advantage when presented with spatially separated target and masker auditory streams, and head movements may partially offset the deficits in spatial hearing commonly observed with sensorineural hearing loss (for review on the effects of aging and hearing loss, see Eddins and Hall, 2010; Gallun and Best, 2020).

Inextricably linked to functioning in complex listening environments is the role of visual information and its interaction with head movements. Using an immersive virtual environment, for example, Hendrikse et al. (2018) measured gaze direction (head-plus-eye movements) in response to multi-talker conversation with and without accompanying video content. Underscoring the importance of the interaction of auditory and accompanying visual information in head movement studies, in the audio-only condition of that experiment, participants did not move their heads at all, in contrast to a variety of head orientations when video was also presented. In a real-time multi-person conversation that quantified head movement, Lu et al. (2021) demonstrated that the head-plus-eye position of the listener was highly predictive of the location of the active talker in a three-way conversation. Together, these experiments highlight the challenges of developing an experimental design that balances control over the variables that elicit head movement, vs. naturalness of the listening environment.

To better understand the role of head movement in complex listening environments and differences between listeners with normal hearing and those with hearing loss, we developed a dual-task experimental paradigm designed to maintain elements of real-world listening by asking participants to simultaneously localize sound and recognize speech, two processes listeners naturally perform while subjected to varying levels of background noise. Specifically, we tested the hypothesis that (1) speech perception would improve with head movement compared to the fixed head condition; (2) the presence of a visual cue at the sound source would further improve speech perception; (3) head movement patterns would differ between the normal hearing and hearing loss groups (e.g., Brimijoin et al., 2010); and (4) hypotheses 1–3 would be modulated by SNR. To accomplish this, we measured head movements during conditions where head movement was fixed to the midline, head movement was free with no visual cue, and when head movement was free plus a visual cue to the sound source location.

## 2 Materials and methods

### 2.1 Participants

**Figure 1:**
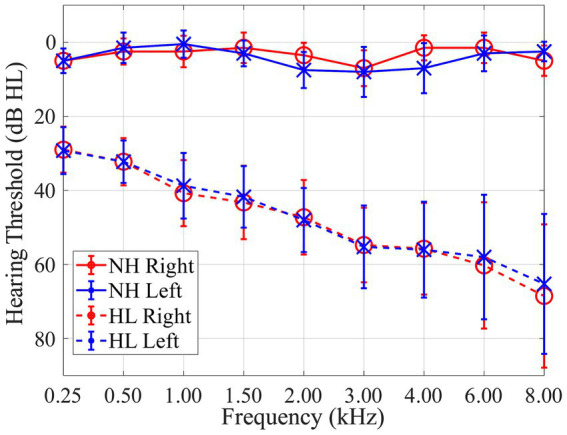
Audiogram showing mean pure tone thresholds with standard deviations for 10 normal-hearing (NH; solid lines) listeners and 20 listeners with hearing-loss (HL; dashed lines) for both right (red) and left (blue) ears in dB HL.

Thirty participants were recruited in this study including 10 normal-hearing (NH) and 20 individuals with hearing-loss (HL). Normal-hearing listeners ranged in age from 22 to 37 years old (mean 26.4; standard deviation 5.5); listeners with hearing loss ranged in age from 52 to 80 years old (mean 70.0; standard deviation 8.3). Based on audiometric evaluation, normal-hearing listeners had pure tone thresholds ≤25 dB hearing level (HL) at octave frequencies between 250 Hz and 8 kHz with ≤10 dB asymmetry between 1 and 6 kHz. Individuals in the hearing loss group had mild-to-severe sensorineural hearing loss (SNHL), stereotypical sloping high-frequency loss, with ≤10 dB asymmetry between 1 and 6 kHz. Mean hearing thresholds with standard deviations of each group are shown in Figure 1. The mean pure-tone average (PTA) for frequencies of 0.5, 1, and 2 kHz was 3 dB HL for both ears for the normal-hearing group and 40 dB HL for both ears for the hearing-loss group. All participants were native English speakers. Participants were excluded if they had recent history (within 6 months) of fluctuating hearing loss, self-reported history of neurological or psychological disorder, or abnormal external ear morphology. All procedures were approved by the University of South Florida Institutional Review Board. Written informed consent was obtained from all participants, and participants were paid for their participation.

### 2.2 Apparatus

**Figure 2:**
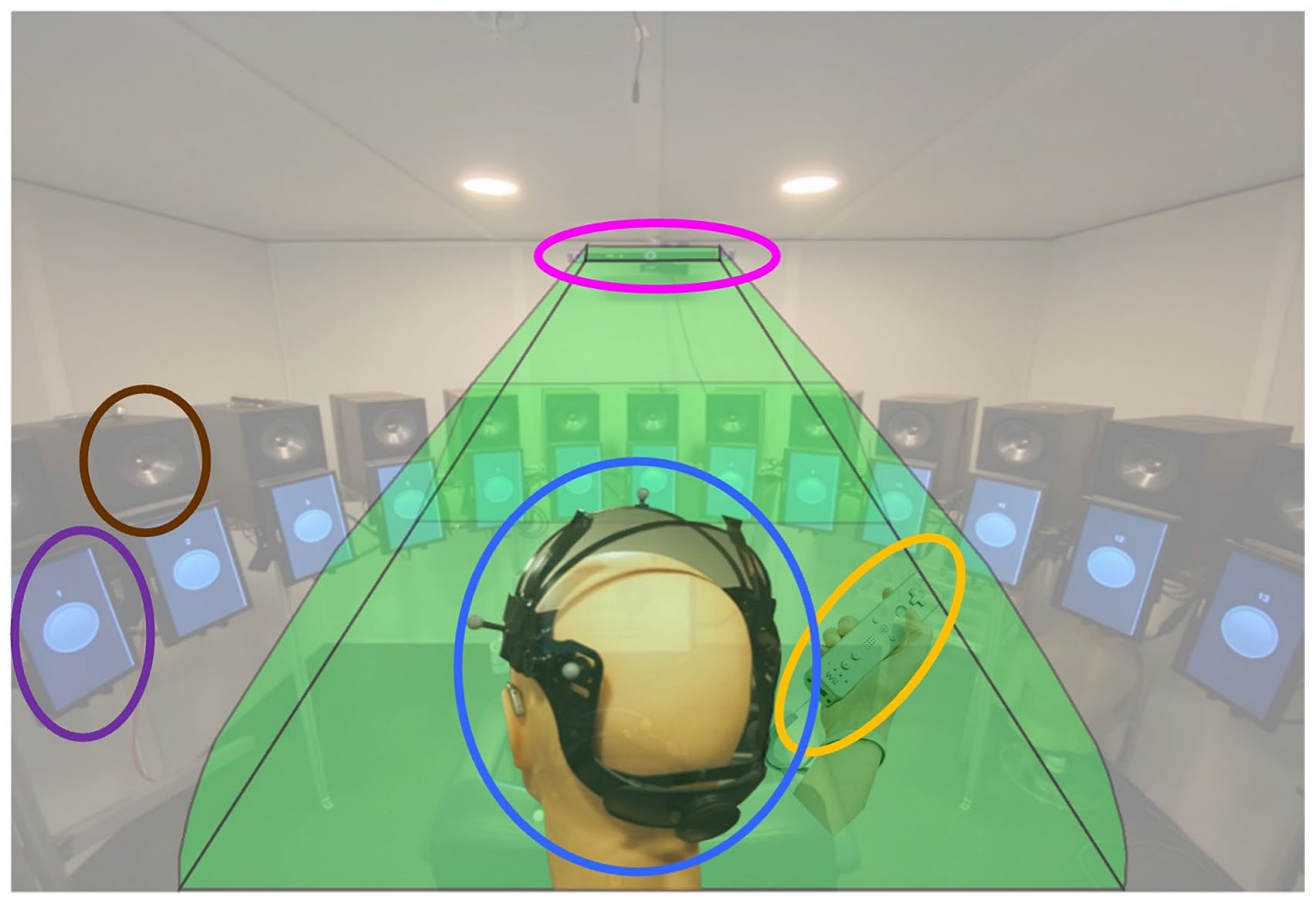
Experiment design. Audio and visual stimuli were presented to a participant seated at the center of the sound-field loudspeaker ring. Head movement was tracked in real time using a head tracking system composed of the head mount placed on the participant head (blue oval) and an infrared motion capture system (OptiTrack V120-Trio, pink oval). Thirteen 11.6″ video monitors (purple oval) are mounted at each loudspeaker (brown oval) location from −90° to +90° azimuth to provide visual cues. Participant’s digit detection response was collected via a Nintendo WiiMote (yellow oval).

During testing, participants were seated in the center of a loudspeaker array consisting of 24 KEF Model Q100 (KEF, London, United Kingdom) loudspeakers separated by 15° (see Figure 2; brown oval) located in a double-walled, sound-attenuating booth (10′ × 10′4″ × 6′6″). To provide visual cues when needed, video monitors (11.6″; Figure 2, purple oval) were mounted below the loudspeaker driver for the 13 loudspeakers located in the front hemifield (i.e., −90° to +90° azimuth). As part of head movement data collection, participants wore an adjustable head band upon which eight reflective spherical markers (Figure 2, blue oval) were attached. Markers were placed in an asymmetric configuration to create a clear distinction between left and right head orientation and avoid marker label confusion. Marker position was monitored using an infrared (IR) motion capture system (OptiTrack Trio V120; NaturalPoint, Inc., Corvallis, OR) suspended from the ceiling in the front-center of the booth (Figure 2, pink oval). Participants indicated detection of a digit by pressing a button on a Nintendo WiiMote (WiiMote; Nintendo Co. Ltd., Kyoto, Japan) (Figure 2, yellow oval, similar to the method of Brimijoin et al., 2013), a device chosen to allow easy and accurate button presses with minimal response-induced head movement.

Raw head-tracking data from the head tracking system were captured with a sampling rate of 120 Hz (120 frames per second, FPS), digitally processed using the manufacturer-provided Motive (OptiTrack) software and streamed to MATLAB in real-time. These values were time-aligned with the stimulus stream and button responses for subsequent offline analyses. Head-tracking data were low-pass filtered to reduce noise and acquisition artifacts. The data acquired from the OptiTrack provided 6 degrees of freedom: 3D translations in the *x*-, *y*-, and *z*-axes (front–back, left–right, and up–down) and rotations around these axes (roll, pitch, and yaw accordingly). Analyses presented here focus on the yaw rotation that represents head turns along the horizontal plane.

### 2.3 Stimuli

The target stream consisted of numbers and other monosyllabic words spoken by a single male talker taken from the Continuous Number Identification Test (CNIT; Ozmeral et al., 2020). Streams were presented from a single spatial location at a rate of ~2 words/s for ~6–8 s. Included numbers were 1–10 excluding the two-syllable digit 7. Each target stream consisted of at least one and up to four numbers interspersed among the other monosyllabic words (inter-word interval = 100 ms). On each trial, target speech streams were pseudo-randomly presented at speaker locations from −90° to 90° in 15° intervals. The CNIT was chosen over comparable speech materials [e.g., coordinate response measure (CRM); Bolia et al., 2000] to meet our experimental goals: it allowed for open-ended testing periods making it less predictable to the participant, and it provided a continuous measure of speech reception rather than designated presentation and response time windows.

Background interferers were played continuously throughout each trial, and consisted of eight-talker in non-English (Spanish, Italian Hungarian, French, Japanese, German, Chinese, and Danish), turn-taking conversations between one female and one male (Ozmeral et al., 2020). Each of the eight interferers was presented from a separate free field location (±15°, ±75°, ±105°, and ±165°). The intensity of the ensemble of background conversations was presented at a fixed level of 60 dB SPL and overlapped temporally with the target stream providing a source of informational masking (see Ozmeral et al., 2020 for spectral content of materials). The target streams were presented at 48-, 54-, 60-, or 66-dB SPL, defining signal-to-noise ratio (SNR) conditions of −12, −6, 0, or + 6 dB. Sound levels were calibrated for the 24-speaker array using free-field protocol with microphone positioned at the location of the participants’ head (i.e., equidistant from each speaker and at the vertical level of the speaker cone).

### 2.4 Tasks and test conditions

#### 2.4.1 Trial initialization

To initiate each trial, participants were required to orient their head to a narrow azimuthal reference location between −0.5° and 0.5° in the yaw domain (monitored by the head tracking system with feedback). When this head orientation was detected, a visual cue (red circle with black background) appeared on the monitor at the 0° position, and simultaneously a speech utterance was presented that said: “Get set to push the button.” Upon the completion of that initial utterance, the red circle was replaced by visual cues corresponding to the head-movement condition, the target stream began, and participants oriented their head according to the specific head-movement condition.

#### 2.4.2 Head-movement conditions

**Figure 3:**
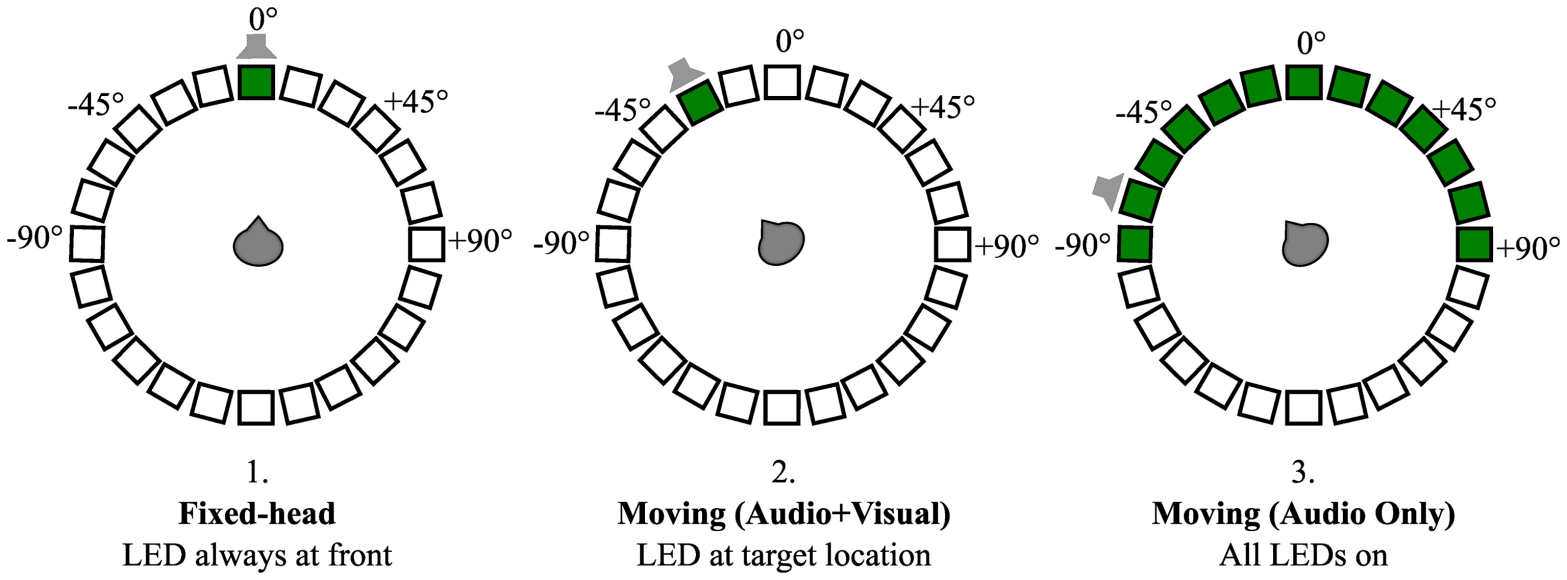
Schematic of three head-movement conditions: (1) Fixed-head, (2) Moving (Audio + Visual), and (3) Moving (Audio Only). Open squares organized in a circle indicate a 24-loudspeaker array with 15-degree separation surrounding participant. Thirteen frontal hemifield loudspeakers had a video-monitor (black boxes) attached to them. Green boxes indicate video-monitor positions where visual cues were presented for condition-specific trials. An example target location is denoted by gray loud-speaker schematic for each condition, illustrating lack of useful information conveyed by the visual cues in the Moving (Audio Only) compared to the Moving (Audio + Visual) condition.

Following trial initialization, a second visual cue consisting of a green circle on a black background was displayed on a condition-specific monitor (position), the onset of this visual cue was synchronized with the onset of the target audio stream. Three conditions were tested (Figure 3): (1) In the Fixed-head, control condition, the visual cue was static and positioned at 0° (front) throughout the trial. Participants were instructed not to move their head away from 0° while responding to the digits regardless of the location of the target stream; (2) In the Moving-head, Audio + Visual condition, the visual cue was positioned at the same location as the target audio stream following the initial utterance (e.g., Best et al., 2007). Participants were instructed to orient their head to the target location as soon as the speech stream started; (3) Moving-head, AudioOnly condition, the visual cue was displayed on all 13 frontal hemifield speaker-monitor locations, such that the cue provided no information about the target location. Participants were nonetheless instructed to orient their head to the location of the audio stream. These head-movement conditions were tested in separate presentation blocks, with 52 trials per block, and the order of blocks was randomized across participant. Within each head-movement condition, four SNRs (−12, −6, 0, and + 6 dB) were tested (three trials for every combination of SNR and test angle) in a counterbalanced order across participants. Participants completed all conditions over the course of 2 to 3 laboratory visits that lasted about 2 h each in duration. Frequent breaks were provided to minimize fatigue and the number of breaks varied among participants.

#### 2.4.3 Digit detection

**Figure 4:**
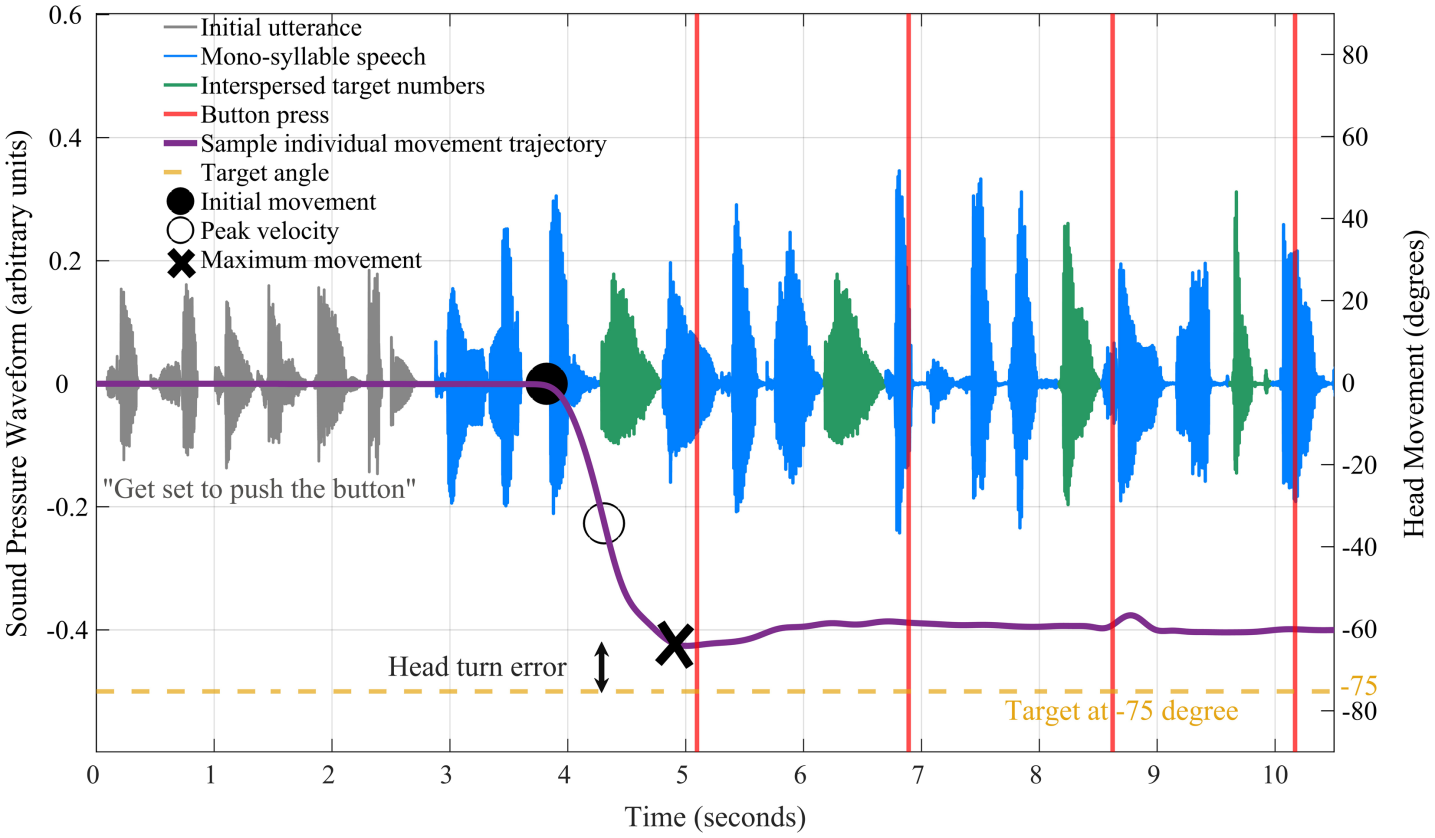
Example time-course data show yaw head-movement trajectory (purple solid line) for a single trial presentation. Audio waveform corresponding to the initial utterance shaded in gray, the mono-syllable speech in blue, and the interspersed target numbers in green. The reference point is noted at 0 degrees and the target in this trial was −75 degrees (yellow dashed line). The target stream began at 0 s followed by the initial head movement (black filled circle), peak velocity (black open circle), and maximum movement (black X marker).

Figure 4 shows an example trial presentation, including the initial utterance when listeners were oriented to the front (gray waveform), the mono-syllable speech (blue), and the interspersed target numbers (green). During the speech stream presentation, regardless of head orientation, listeners indicated when they heard a spoken number by pressing the response button in their dominant hand (Figure 4; red vertical lines). Responses were considered as correct detections when the button was pressed within 1-s following the onset of digit presentation.

### 2.5 Analysis of head movement

In addition to digit detection accuracy, the following features were extracted from head movements (“yaw trajectory”; Figure 4; purple solid line): (1) starting time of initial movement relative to the start of the speech stream (“temporal coherence”; Figure 4; black filled circle marker); (2) “peak velocity” of the head movement (Figure 4; black open circle marker); (3) the peak head displacement in the yaw trajectory relative to the 0° starting point (“maximum movement”; Figure 4; black “X” marker); (4) the absolute discrepancy between head orientation and target speech location (“head turn error”). Time to initial head movement was defined as the first point to exceed 5% of the rms of head displacement calculated over the first 50 ms of the yaw trajectory (i.e., relative to baseline).

All statistical analyses were conducted using IBM SPSS Statistics (Version 26). Greenhouse–Geisser (when estimated epsilon was less than 0.75) and Huynh Feldt (when estimated epsilon was greater than 0.75) corrections were used when there was a violation of sphericity. Partial eta squared and Cohen’s *d* are also reported indicating the effect sizes.

## 3 Results

### 3.1 Digit detection accuracy

**Figure 5:**
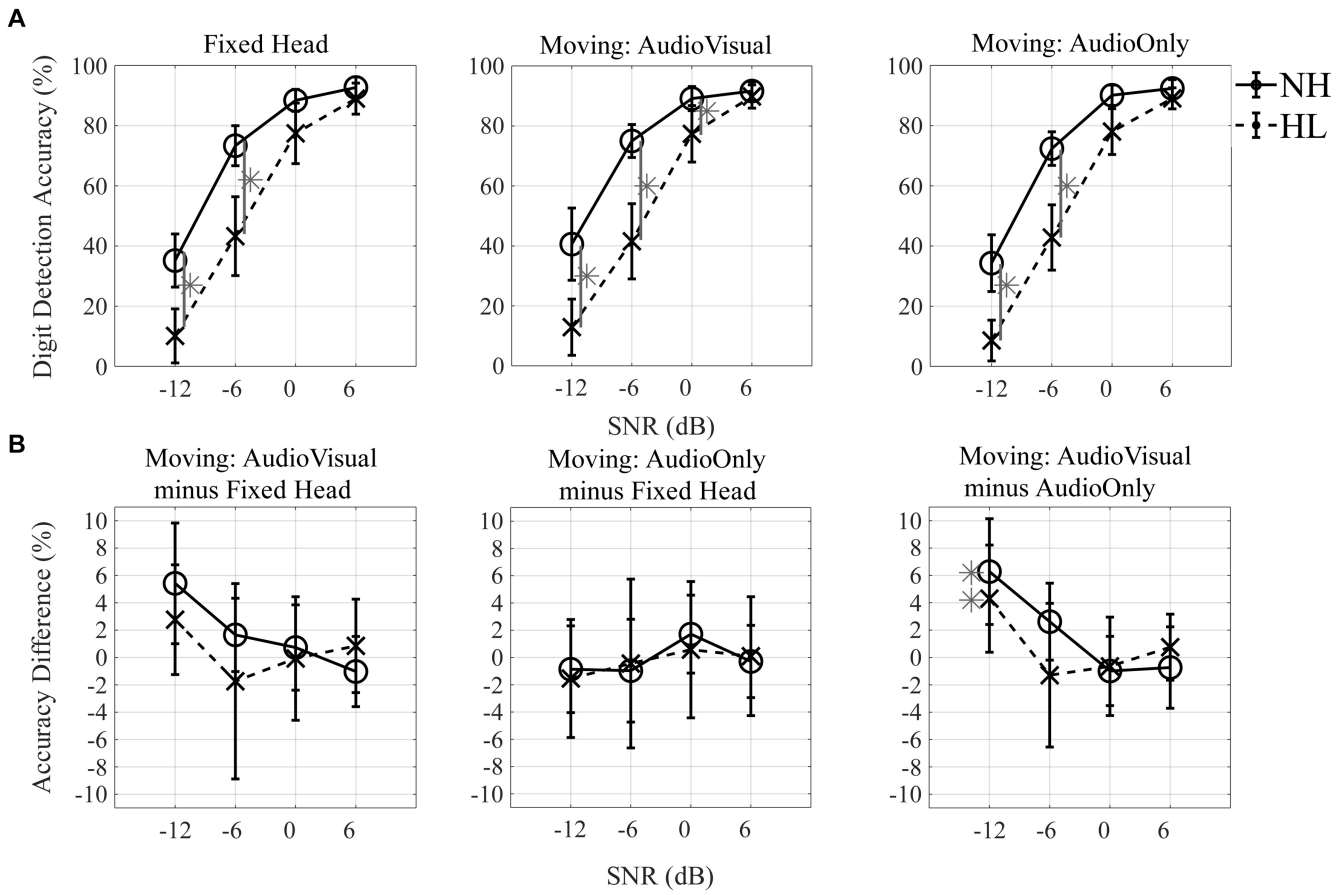
Digit detection accuracy and accuracy difference. **(A)** Digit detection accuracy (%) across four different SNRs (−12, −6, 0, and + 6 dB) for three head-movement conditions [Fixed-head, Moving (Audio + Visual), and Moving (Audio Only)]. **(B)** Accuracy difference (%) between the fixed condition and each of the two moving conditions across SNRs (first and second columns) and between the two moving conditions across SNRs (third column). Mean and standard error of the mean plotted for normal-hearing listeners (solid lines with circle markers), and individuals with hearing loss (dashed lines with X markers). Asterisks indicate significant difference of *post-hoc* paired *t*-test at the 0.05 level **(A)** and significant difference of *post-hoc* one-sample *t*-test comparing to zero at the 0.05 level **(B)**.

Digit detection accuracy was assessed by computing the number of the correctly detected digits divided by the total number of presented digits for each trial. Initial statistical analysis included spatial location of the target stream (13 levels) as a factor in ANOVA testing. As there was no significant effect of angle, detection accuracy for all subsequent statistical tests was averaged across all 13 target-angles for each condition and group. An additional follow-up test verified that no significant differences were observed for digit detection performance between the four speaker locations that also presented the background masker compared to the nine locations that did not. The resulting data are plotted in Figure 5A with SNR on the *x*-axis and digit detection accuracy on the *y*-axis with the three head-movement conditions in separate panels from left to right. Data for the normal-hearing group are indicated with open circles (solid line) and data for the hearing-loss group are shown by “*x*” symbols (dashed lines). Results of a repeated-measures ANOVA with two within-subject factors (three levels of head-movement condition, and four levels of SNR) and one between-subject factor (two groups) showed a main effect of SNR; as SNR increased, digit detection accuracy increased (*F*_2.1,58.9_ = 228.64, *p* < 0.001; η_p_^2^ = 0.891). There was a significant main effect of group (*F*_1,28_ = 12.95, *p* = 0.001; η_p_^2^ = 0.316), with higher accuracy for the normal-hearing listeners than the hearing-loss group. The SNR effect was stronger for the hearing-loss group than the normal-hearing group, an effect reflected in the significant interaction between SNR and group (*F*_2.1,58.9_ = 10.43, *p* < 0.001; η_p_^2^ = 0.271). The head-movement condition effect was not significant (*F*_2,56_ = 0.74, *p* = 0.484, η_p_^2^ = 0.026), but there was a significant interaction between head-movement condition and SNR (*F*_6,168_ = 2.23, *p* = 0.043; η_p_^2^ = 0.074). This interaction is best explained by the fact that the difference between the two groups was greatest in the −12 dB SNR and − 6 dB SNR conditions, and with increasing SNR greater similarity was observed between groups, approaching 90% detection accuracy for all head-movement conditions at +6 dB for both groups (Figure 5A). A three-way interaction between head-movement condition, SNR, and group was not significant (*F*_6,168_ = 0.53, *p* = 0.785, η_p_^2^ = 0.019). *Post-hoc* paired *t*-tests were conducted across conditions and gray asterisks shown in Figure 5A indicate significant differences between groups at corresponding SNRs (*p* < 0.05 level).

To evaluate whether performance improved when participants were allowed to move their heads during the digit detection task, the difference in performance accuracy between the fixed-head condition and each of the two moving conditions was computed, a value termed the “accuracy difference” (Figure 5B). Head-movement advantages all were within ±0.1 (10%), indicating only minor head-movement benefit for these conditions. No significant differences were observed between the fixed and either moving-head conditions (Figure 5B; left and middle panels). Based on one-sample *t*-tests (at the *p* < 0.05 level of significance), the accuracy difference between the Moving (Audio+Visual) and Moving (Audio Only) (Figure 5B; right panel) was significantly different from zero at −12 dB SNR for both normal-hearing and individuals with hearing loss (normal-hearing participants: *t*_9_ = 2.6, *p* = 0.030, *d* = 0.81, 95% confidence interval 0.75–11.82; individuals with hearing loss: *t*_19_ = 2.5, *p* = 0.024, *d* = 0.55, 95% confidence interval 0.64–7.97).

### 3.2 Head orientation

**Figure 6:**
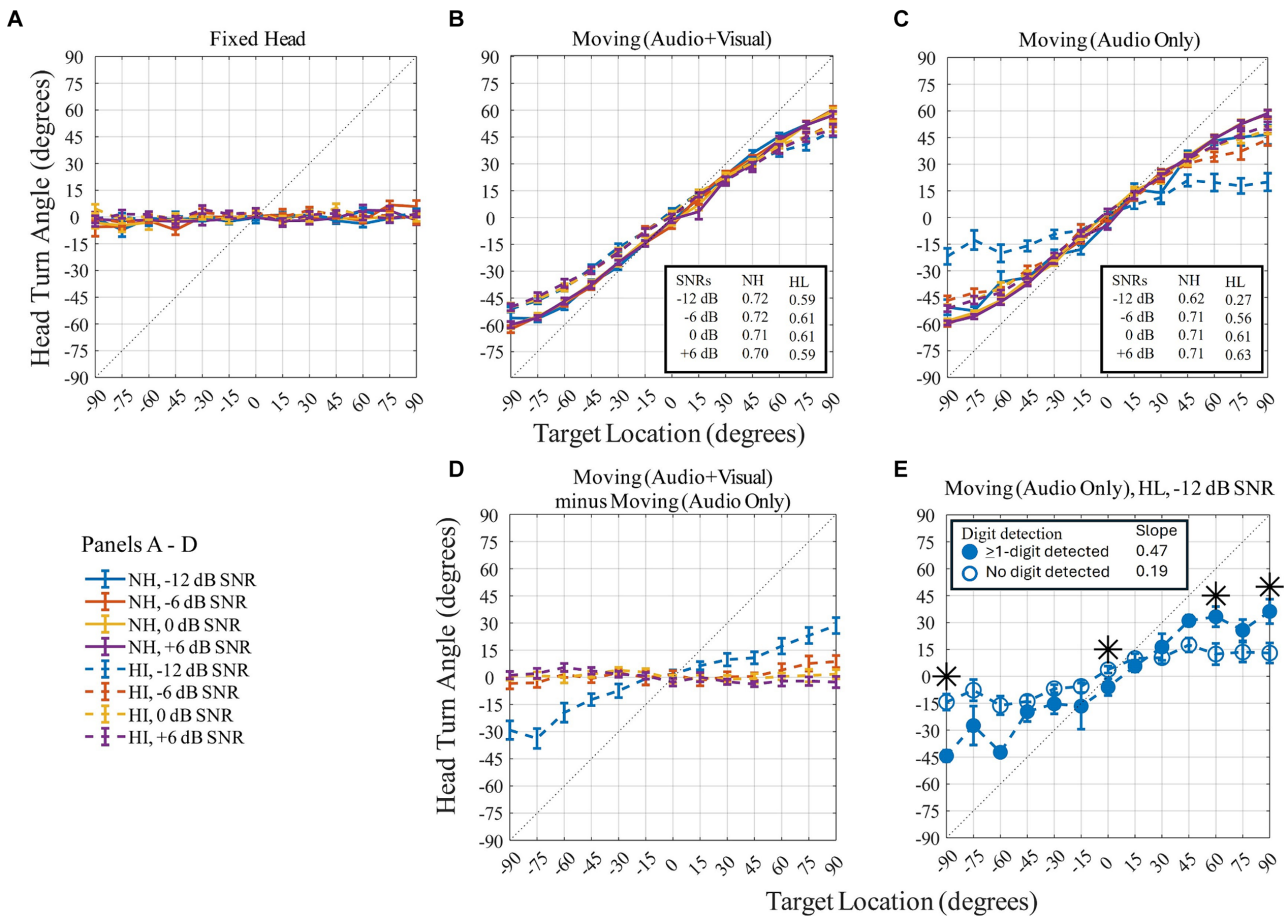
Panel **(A–C)** show peak head turn angle (in degrees) across target locations (in degrees) on three head-movement conditions. Panel **(D)** quantifies the difference between the two moving conditions. Normal-hearing group indicated with solid lines, hearing-loss group with dashed lines. Colors differentiate level of dB SNRs. In-figure texts indicate slopes for each group at each SNR. Panel **(E)**: head turn angle (in degrees) for the −12 dB SNR of the hearing-loss group during the Moving (Audio Only) condition plotted as a function of target location (in degrees). Symbols indicate condition: open blue circles for no-digit detected trials and filled blue circles for ≥1-digit detected trials. Symbols correspond to the mean; error bars to standard error of mean. Dotted line represents ideal match between head orientation and target location. Asterisks denote target locations with a significant difference between head turn angle when 0-digit was detected compared to trials where at least 1-digit was detected (paired *t*-test).

Head orientation was quantified using multiple features including the maximum head displacement in the yaw-trajectory relative to the 0° starting point for each trial (Figure 6). Figure 6 shows the maximum (peak) head turn angle (in degrees) on the *y*-axis as a function of the 13 target locations (in degrees) on the *x*-axis for the three head-movement conditions (Figures 6A–C) and for the difference between two moving conditions (Moving (Audio+Visual) minus Moving (Audio Only); Figure 6D). The flat response in Figure 6A confirms that participants maintained their head orientation to the front during the Fixed-head, control condition. For the Moving (Audio+Visual) condition, a strong positive correlation between target angle and head angle was observed. This relationship had a slope less than 1, reflecting an undershoot of the head angle relative to the target angle. For example, when the target was at 90°, the maximum head turn angle on average was about 60°, representing a 30° undershoot. Slopes for each SNR are reported for the two moving head conditions in the insets for each of the corresponding panels in Figures 6B,C. For the hearing-loss group, the undershoot effect was most pronounced [with an average-across-SNR slope of 0.60, *r*^2^ = 0.997, *p* < 0.001, for the Moving (Audio+Visual) and 0.52, *r*^2^ = 0.995, *p* < 0.001, for the Moving (Audio Only)]. Participants in the hearing-loss group tended to turn their heads less toward the target location than did participants in the normal-hearing group [with an average-across-SNR slope of 0.71, *r*^2^ = 0.997, *p* < 0.001, for the Moving (Audio+Visual) and 0.69, *r*^2^ = 0.997, *p* < 0.001, for the Moving (Audio Only)].

For the two moving conditions only, a repeated-measures ANOVA was performed with three within-subject factors (two levels of head-movement condition, four levels of SNR, and 13 levels of target angles) and one between-subject factor (two groups). Different patterns of results were observed for the head-moving conditions with and without the visual cue. The SNR and angle effects were most pronounced and had a significant main effect. There were also significant main effects of group and significant two- and three-way interactions (shown in Table 1). These results were primarily driven by the lack of movement exhibited by the hearing-loss group in the −12 dB SNR condition, where head angle was limited to the −30 to +30-degree span (Figure 6C, blue dashed line). To further investigate the group differences in this −12 dB SNR condition, we performed two additional analyses. The first quantified the head movement difference with the visual cue vs. without the visual cue (as shown in Figure 6D). Apart from the −12 dB SNR condition for the hearing-loss group, all other difference-plots are essentially flat, indicating similarity in the range of maximal head movement across the range of source locations. The second follow-up analysis split the dataset for the group with hearing loss at −12 dB SNR (Figure 6E) into two sets of trial types: (1) trials where no digits were detected (open circles), and (2) trials where at least one digit was detected (closed circles). The results indicate significantly greater head movement to lateral targets when a digit was detected than when a digit was not detected. An independent sample *t*-test between the two trial types was performed. Asterisks at the target locations (−90°, 0°, 60°, 90°) indicate where there was significant difference in mean head turn angle between the no-digit detected trials and the ≥1-digit detected trials (at −90°: *t*_17_ = −5.5, *p* < 0.001, *d* = −1.93; at 0°: *t*_18_ = −2.2, *p* = 0.042, *d* = −1.22; at 60°: *t*_18_ = 2.3, *p* = 0.035, *d* = 1.07; at 90°: *t*_18_ = 2.4, *p* = 0.028, *d* = 1.17). Lastly, the undershoot effect was significantly more pronounced for the no-digit detected trials (with a slope of 0.19, *r*^2^ = 0.921, *p* < 0.001) than the ≥1-digit detected trials (with a slope of 0.47, *r*^2^ = 0.968, *p* < 0.001).

**Table 1:** Repeated-measures ANOVA based on head turn angle averaged from all digits of the two moving conditions.

Effect	*F*_df_	*p* value	η_p_^2^
Head-movement condition	0.37_1,26_	0.549	0.014
SNR	0.77_2.1,54.8_	0.473	0.029
Angle	1080.11_1.8,46.0_	<0.001**	0.976
Group	2.50_1,26_	0.126	0.088
Head-movement condition × Group	2.56_1,26_	0.122	0.090
SNR × Group	0.89_2.1,54.8_	0.421	0.033
Angle × Group	13.82_1.8,46.0_	<0.001**	0.347
Head-movement condition × SNR	0.49_2.6,68.7_	0.669	0.018
Head-movement condition × Angle	4.42_2.8,72.1_	0.008	0.145
SNR × Angle	8.68_36,936_	<0.001**	0.025
Head-movement condition × SNR × Group	2.29_2.6,68.7_	0.094	0.081
Head-movement condition × Angle × Group	1.44_2.8,72.1_	0.240	0.053
SNR × Angle × Group	3.86_36,936_	<0.001**	0.129
Head-movement condition × SNR × Angle	9.29_36,936_	<0.001**	0.263
Head-movement condition × SNR × Angle × Group	3.19_36,936_	<0.001**	0.109

**denote significant effects with at the 0.001 alpha-level.

**Figure 7:**
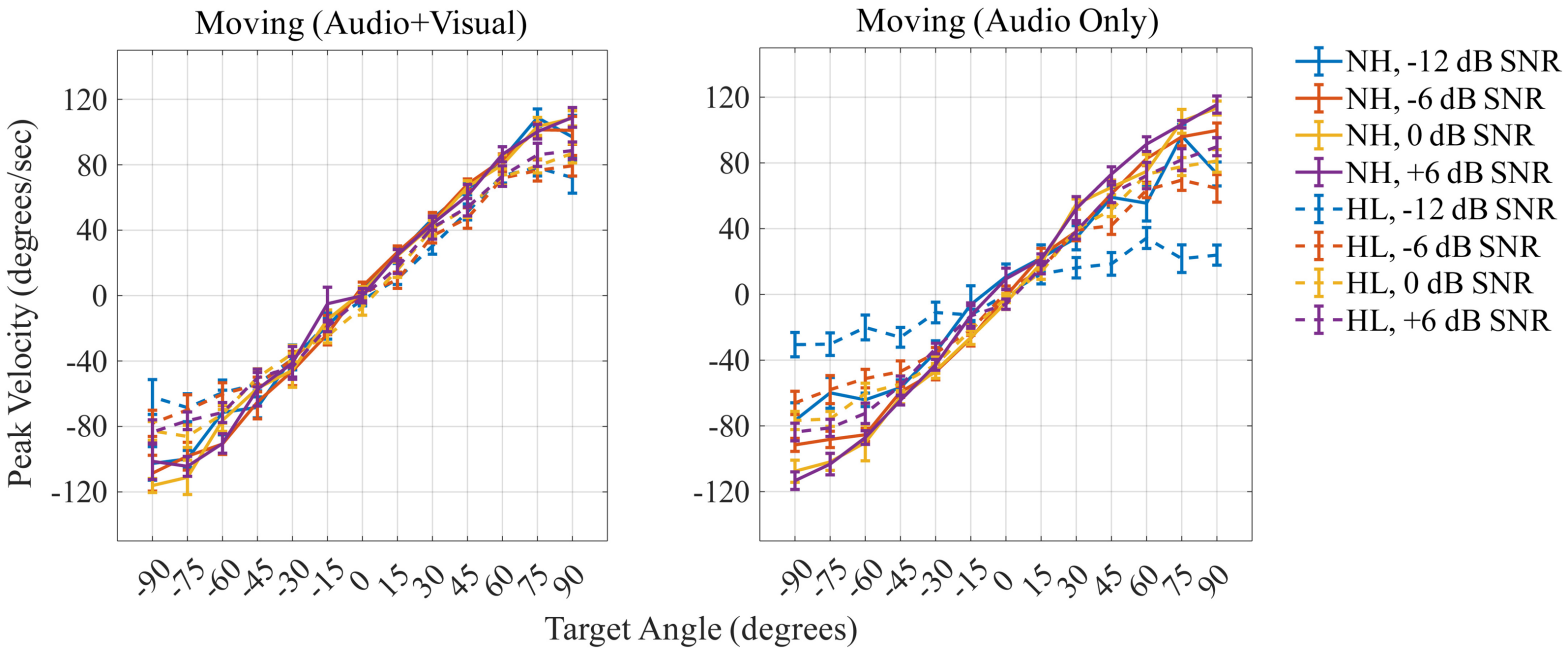
Peak velocity (in degrees/s) across target locations for the two moving conditions (Moving: Audio + Visual; left panel, AudioOnly; right panel). Positive values indicate peak velocity to the right, negative values to the left, for normal-hearing (solid lines) and hearing-loss (dashed lines) groups. Colors differentiate the level of dB SNRs. In-figure texts denote slopes for each group at each SNR.

Figure 7 shows the peak velocity (in degrees/s) on the *y*-axis as a function of the 13 target angles (in degrees) on the *x*-axis for the two moving conditions [Moving (Audio+Visual); left panel, Moving (Audio Only); right panel]. A repeated measures ANOVA was performed with three within-subject factors (two levels of head-movement condition, four levels of SNR, and 13 levels of target angles) and one between-subject factor (two groups). The results are reported in Table 2. Peak velocity of head turns was significantly affected by SNR (*F*_3,78_ = 3.03, *p* = 0.034, η_p_^2^ = 0.104); peak velocity increased as SNR increased. There was also a significant effect of angle (*F*_1.7,43.3_ = 449.89, *p* < 0.001, η_p_^2^ = 0.945); participants reached the highest velocity at 120 degrees/s when the target was at +90° or −90°. Peak velocity during the Moving (Audio+Visual) and Moving (Audio Only) were not significantly different (*F*_1,26_ = 0.14, *p* = 0.709, η_p_^2^ = 0.005), and there was also no significant main effect of group (*F*_1,26_ = 0.48, *p* = 0.493, η_p_^2^ = 0.018).

**Table 2:** Repeated-measures ANOVA based on peak velocity averaged from all digits of the two moving conditions.

Effect	*F*_df_	*p* value	η_p_^2^
Head-movement condition	0.14_1,26_	0.709	0.005
SNR	3.03_3,78_	0.034*	0.104
Angle	449.89_1.7,43.3_	< 0.001**	0.945
Group	0.48_1,26_	0.493	0.018
Head-movement condition × Group	0.00_1,26_	0.971	0.000
SNR × Group	1.25_3,78_	0.298	0.046
Angle × Group	9.85_1.7,43.3_	< 0.001**	0.275
Head-movement condition × SNR	0.10_3,78_	0.962	0.004
Head-movement condition × Angle	4.47_4.5,117.7_	0.001*	0.147
SNR × Angle	10.62_36,936_	< 0.001**	0.290
Head-movement condition × SNR × Group	1.05_3,78_	0.376	0.039
Head-movement condition × Angle × Group	1.24_4.5,117.7_	0.296	0.046
SNR × Angle × Group	1.66_36,936_	0.010*	0.060
Head-movement condition × SNR × Angle	6.50_36,936_	< 0.001**	0.200
Head-movement condition × SNR × Angle × Group	1.32_36,936_	0.098	0.048

Asterisks denote significant effects at the 0.05 (*) and 0.001(**) alpha-level.

### 3.3 Dual-task temporal coherence

**Figure 8:**
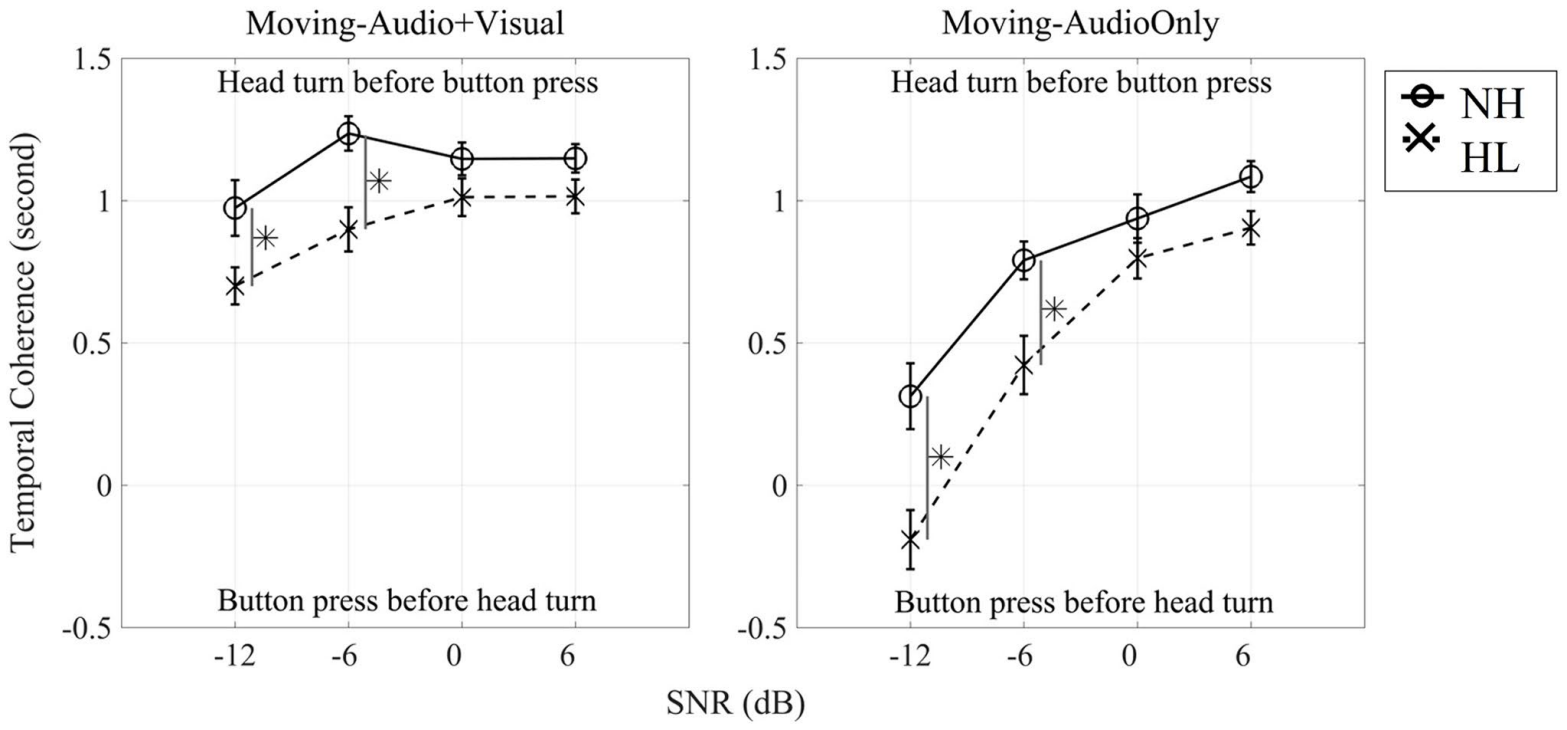
Dual-task temporal coherence between button presses and the initiation of head movements averaged from all 13 target-locations for each SNR for the Moving (Audio + Visual) condition (left panel) and for the Moving (Audio Only) condition (right panel). Solid line indicates normal-hearing listeners whereas dashed line indicates hearing-loss listeners. Asterisks denote significant differences at the 0.05 level, Bonferroni corrected.

Given the dual-task design of this study, in which listeners performed digit detection and speech localization simultaneously, it was of interest to know whether the tasks were carried out in a stereotypic manner among listeners and across listener groups. For example, was one task prioritized over the other? We examined the temporal dynamic between tasks by computing a time lag between a head-turn onset and the first button press. This was defined as the head-turn onset time minus the first button-press time and termed “temporal coherence.” Temporal coherence (in seconds) for each of the Moving conditions is presented in Figure 8. Initial statistics revealed no effect of target angle, therefore figures and statistical tests represent the dataset averaged across the 13 target locations. Positive values of coherence indicate that the head turn occurred before the button press, whereas negative values of coherence indicate that the listener executed the button press before moving their head.

Figure 8 shows that for the Moving (Audio+Visual) condition, both normal-hearing and hearing-loss groups turned their head before pressing the button for all four SNRs. To determine whether temporal coherence (averaged temporal coherence across 13 locations) differed across the four SNRs, a three-way, repeated measures ANOVA with two within-subject factors (three levels of head-movement condition, and four levels of SNR) and one between-subject factor (two groups) was computed (Figure 8 left panel and Table 3). There was a significant main effect of head-movement condition (*F*_1,28_ = 80.40, *p* < 0.001, η_p_^2^ = 0.742). When the visual cue was provided, both groups on average turned their heads to the visual target before pressing a button. There was also a significant main effect of SNR (*F*_2.1,57.4_ = 77.08, *p* < 0.001, η_p_^2^ = 0.734), typified by faster initiation of head turn with easier (positive) SNR conditions. As the SNR decreased, the time lag between head turns and button press decreased. There also was a significant main effect of group (*F*_1,28_ = 8.71, *p* = 0.006, η_p_^2^ = 0.237); normal-hearing listeners had higher temporal coherence than individuals with hearing loss. However, two-way interactions of SNR × group and head-movement condition × SNR were also significant (SNR × Group: *F*_2.1,57.4_ = 4.69, *p* = 0.012*, η_p_^2^ = 0.144; head-movement condition × SNR: *F*_3,84_ = 24.69, *p* < 0.001, η_p_^2^ = 0.469). The first interaction can be explained by the generally larger difference in temporal coherence between the groups at −12-dB and − 6-dB SNRs vs. the higher SNRs. The second interaction is explained by the drastically different slopes of the lines between the two Moving conditions, one in which the visual cue was provided (left panel), and therefore, listeners were more likely to move early and before their first button press. There was no three-way interaction.

**Table 3:** Repeated-measures ANOVA based on temporal coherence (based on Figure 8).

Effect	F_df_	P-value	η_p_^2^
Head-movement condition	80.40_1,28_	<0.001**	0.742
SNR	77.08_2.1,57.4_	<0.001**	0.734
Group	8.71_1,28_	0.006*	0.237
Head-movement condition × Group	0.83_1,28_	0.371	0.029
SNR × Group	4.69_2.1,57.4_	0.012*	0.144
Head-movement condition × SNR	24.69_3,84_	<0.001**	0.469
Head-movement condition × SNR × Group	0.71_3,84_	0.551	0.025

## 4 Discussion

The goal of this study was to investigate head-orienting behaviors of listeners with normal hearing and hearing loss in the azimuthal plane with and without accompanying visual information under a set of well-controlled conditions, and to provide metrics of head movement in varying listening environments. Participants were instructed to (1) detect meaningful speech targets (numbers) via button press and (2) localize via head movement to a target stream of monosyllabic words. This dual-task paradigm was designed to characterize the patterns of head movement individuals use in difficult listening environments. One of our predictions was that head movement compared to the fixed-head condition would significantly improve performance on the digit detection task. While this general hypothesis was not proven, we did see a significant improvement when participants were visually cued to the target location (Figure 5B, right panel). This result was observed for both normal and hearing loss groups. Another expectation was that that individuals with hearing loss would have different head-movement behaviors when localizing speech in noise compared to normal-hearing listeners, with the notion that the two groups likely are accustomed to differences in the ability to use acoustic spatial cues or differences in the weighting of those cues. The results showed significant main effect of SNR and group on the digit detection accuracy for all three head-movement conditions. Finally, we observed that when no visual spatial cue was provided in the most difficult listening environment, individuals were slower to initiate head movements, opting instead to hold still, listen, and then move.

### 4.1 Digit detection accuracy

In contrast to Grange and Culling (2016a), the current study revealed only minor head-orienting benefits of within a range of ±10% of digit detection accuracy, represented by the accuracy difference (Figure 5B). Multiple differences between the two studies could explain the discrepancy, starting with the general experimental paradigm. For one, the masker types and masker locations were quite different; Grange and Culling presented speech-shaped-noise maskers from a single back-hemifield loudspeaker located at either 97.5°, 112.5°, 150°, or 180°, while the current study effectively presented a diffuse masker, consisting of continuous, two-talker, turn-taking conversational sources simultaneously from 8 loudspeaker locations (±15°, ±75°, ±105°, ±165°). Another experimental difference was target location: Grange and Culling (2016a) presented the target speech from 0° and the current study presented the target stream at pseudo-random positions between −90° and 90° in 15° intervals. Finally, Grange and Culling (2016a) calculated head-orientation-benefit based on a procedure that required participants to repeat back a certain number of words from the target speech, and the SNR of the masker was adapted to reach their speech-reception-threshold. In our experiment, digit detection accuracy was used to define the benefit of head-movement. One interpretation of these two studies juxtaposed is that the spatial configuration and masker types affect spatial-release from masking in different ways, leading to differing estimates of head-orientation-benefit. In other words, while the Grange and Culling study quantified the spatial release from masking benefit provided by head orientation, our results show that head movement, even when guided by a visual cue, and in conditions where no better ear advantage is available, does not provide substantial benefit. Furthermore, by presenting target speech from a wide range (13 locations), our study introduced a substantial uncertainty for our listeners that made it especially difficult at low SNRs, providing another likely explanation for differences in measurable benefit of head movement. Lastly, when the target was fixed to the front, as in Grange and Culling, orienting the head away from the front creates a greater release from masking compared to when the head is oriented toward a target at the side (Hawley et al., 2004; Jelfs et al., 2011). Taken together, all these differences likely contribute to the discrepancy in head orientation benefit of up to 8 dB observed by Grange and Culling vs. the 1.7 and 1.2 dB observed in the current study for normal-hearing and hearing-loss listeners, respectively (Figure 5B).

### 4.2 Head orienting behaviors

Consistent with previous investigations, the current study demonstrated that target location, SNR, and hearing impairment have varying impacts on head orienting behaviors. Typically, normal-hearing listeners and individuals with hearing loss undershoot auditory targets when orienting their heads in the direction of target source (Brimijoin et al., 2010; Hládek et al., 2019; Lewald et al., 2000; Lu et al., 2021). The yaw orientations observed here were typically within a range of ±60 degrees for our range of ±90-degree targets, well described as a linear relationship with slopes around 0.6–0.7 in the >0 dB SNR conditions. These values agree with previous studies in laboratory-based, speaker-array environments (Brimijoin et al., 2010) as well as conversational real-world type environments (Lu and Brimijoin, 2022).

Brimijoin et al. (2010) also observed a significant correlation between degree of hearing loss and the root-mean-squared (RMS) difference in degrees between visual and auditory fixation. As hearing threshold increased, the undershoot of auditory targets for individuals with hearing loss was less than visual targets. Their interpretation was that listeners with hearing loss were likely to compensate for greater head movement undershoot using eye gaze to further orient toward the sound source. In addition, Hendrikse et al. (2019) used an experimental paradigm that measured head-gaze ratio in young normal hearing and older normal-hearing listeners in realistic virtual environments. They reported that the older listeners had a significantly higher ratio, meaning they moved their heads relative to their eyes more than younger normal-hearing listeners. Though it is difficult to draw a straight comparison between Hendrikse et al. (2019) and the current study, notably due to difference in task-instructions (move naturally vs. move to the target), a reasonable expectation would be for greater head movement in the hearing-loss group compared to the normal-hearing group. The opposite of this expectation was observed, with the hearing-loss group exhibiting less head movement, particularly in the most difficult listening environments. The most likely explanation for this deviation is the experimental paradigm and the dual-task design. Rather than providing listeners with a video representation of a talker (as in Hendrikse et al., 2019), the visual cue only provided spatial information; once known, there was no additional benefit to the listener. In the case of Brimijoin et al. (2010), the assigned task was to point their nose to the target and hold it there, compared to our instructions to detect digits in the target speech, and move their head to that location.

The condition with the largest head orientation error was observed for the group with hearing loss during the −12-dB SNR, Moving (Audio Only) condition, with head orientations ranging from −30° to +30° on average (Figure 6C, blue line). As this is the most difficult condition and based on digit detection performance (Figure 5A, panel 3), it is a fair interpretation to say that these listeners had great difficulty finding the target stream and could not orient their head to the target. To test this interpretation, we split the head orientation dataset for the hearing-loss group at −12 dB SNR into trials where no digits were detected (Figure 6E, circles) and trials where at least one digit was detected (Figure 6E, triangles). Indeed, this analysis showed that head-turn angle was comparable to easier listening conditions at high SNRs when at least one digit was detected. This analysis might also be framed with a null hypothesis that when no digits were detected, the location of the target stream was unknown to the listener, and head orientation as a function of target location should be flat. This hypothesis is disproven, however, as head orientation during these trials (Figure 6E, circle) has a slope of 0.19 (*r*^2^ = 0.921, *p* < 0.001), indicating that there is information about the hemifield of the target stream embedded in the head orientation, even when the digit detection task could not be performed.

### 4.3 The time to initiate head movement

In the dual-task paradigm, participants were instructed to perform both digit detection and target stream localization without instructions to prioritize either task. Temporal coherence between these tasks was used to quantify which task listeners prioritized by comparing the time of initial head movement relative to the time of first button press. Results showed that temporal difference was faster by approximately 1-s, for head turn initiation during high SNRs and when the visual target was available [i.e., Moving (Audio+Visual) condition], while at lower SNRs, the hearing-loss group was a little slower to turn their head to the target stream compared to normal-hearing listeners (Figure 8, left panel). The interpretation here is that listeners detected the visual cue and oriented their heads at once and then pressed the button when a digit was detected. At difficult SNRs without the visual cue however, the hearing-loss group in particular delayed orientation. In the Moving (Audio Only) condition, temporal coherence for both groups approached 0 s during the low-SNR conditions (Figure 8, right panel). One interpretation of this pattern is that when no visual cue was provided and the target stream was not easily located, listeners prioritized holding still at 0° and gathering information rather than performing a head movement searching behavior. In the easier listening conditions participants located the target stream, turned their head, and then when a digit was presented, responded. In the more difficult conditions that target stream was still available (Figure 4), but rather than orient the head to it, they waited to hear a digit. In other words, it appears that listeners prioritized the digit detection task over the localization task in the most difficult listening conditions, a behavior that may reflect a general listening strategy that prioritizes identifying target-meaning over target-location. This result is consistent with those of Brimijoin et al. (2010) who showed a correlation between pure-tone average hearing threshold and delayed initial latency to orient to the target. The results presented here fill in two important gaps: (1) the individual prioritized the content of the target stream over orientation to target location, and (2) this pattern was observed in both normal hearing and hearing loss groups. Future studies may explore this trade off in more depth.

## 5 Conclusion

In conclusion, this study demonstrated that as head-turn error decreased, digit detection accuracy increased, and that visually-cued head orientation toward an auditory target stream provided a small but significant benefit to speech detection. In the most difficult listening conditions (i.e., lowest SNR values) in the AudioOnly condition (without a visual cue to target location), the benefit of head-movement disappeared. In all freely moving head conditions, head orientation was linearly related to target location with a consistent undershoot relative to the target at extreme angles. The primary deviation from this pattern was noticed among listeners with hearing loss at the low-SNR conditions. Subsequent analyses of head orientation during trials where target digits were undetected revealed hemifield sensitivity to the target stream that was sub-threshold to digit detection. Temporal coherence between initial head movement to digit detection showed a behavioral pattern that prioritizes information gathering (and holding still) during difficult listening conditions rather than an active listening search strategy. In summary, the dual-task paradigm offers a valuable perspective on the head movement and listening strategies human listeners use when faced with listening environments of varying difficulty. Future investigations should address individualized patterns of head movement such as pitch and roll and dynamics as they relate to communication actions, intentions, and specific environmental conditions.

## Data Availability

The raw data supporting the conclusions of this article will be made available by the authors, without undue reservation.
